# Intramedullary Spinal Cord Abscess with Concomitant Spinal Degenerative Diseases: A Case Report and Systematic Literature Review

**DOI:** 10.3390/jcm11175148

**Published:** 2022-08-31

**Authors:** Redwan Jabbar, Bartosz Szmyd, Jakub Jankowski, Weronika Lusa, Agnieszka Pawełczyk, Grzegorz Wysiadecki, R. Shane Tubbs, Joe Iwanaga, Maciej Radek

**Affiliations:** 1 Department of Neurosurgery, Spine and Peripheral Nerves Surgery, Medical University of Lodz, 90-549 Lodz, Poland; 2 Department of Clinical Chemistry and Biochemistry, Medical University of Lodz, 90-419 Lodz, Poland; 3 Department of Normal and Clinical Anatomy, Chair of Anatomy and Histology, Medical University of Lodz, Żeligowskiego 7/9, 90-752 Lodz, Poland; 4 Department of Neurosurgery, Tulane Center for Clinical Neurosciences, Tulane University School of Medicine, New Orleans, LA 70112, USA; 5 Department of Neurosurgery and Ochsner Neuroscience Institute, Ochsner Health System, New Orleans, LA 70433, USA; 6 Department of Neurology, Tulane Center for Clinical Neurosciences, Tulane University School of Medicine, New Orleans, LA 70112, USA; 7 Department of Anatomical Sciences, St. George’s University, Grenada FZ 818, West Indies; 8 Department of Surgery, Tulane University School of Medicine, New Orleans, LA 70112, USA

**Keywords:** intramedullary spinal cord abscess (ISCA), abscess, spinal cord, laminectomy, myelotomy

## Abstract

Intramedullary spinal cord abscess (ISCA) is a rare clinical pathology of the central nervous system that usually accompanies other underlying comorbidities. Traditionally it has been associated with significant mortality and neurological morbidities because it is often difficult to diagnose promptly, owing to its nonspecific clinical and neuroimaging features. The mortality rate and the outcome of these infections have been improved by the introduction into clinical practice of antibiotics, advanced neuroimaging modalities, and immediate surgery. We report the case of a 65-year-old male patient who presented with a progressive spastic gait and lumbar pain, predominantly in the left leg. An MRI image revealed an expansile intramedullary cystic mass in the thoracic spinal cord, which was initially diagnosed as a spinal tumor. He underwent laminectomy and myelotomy, and eventually the pus was drained from the abscess. The follow-up MRI showed improvement, but the patient’s paraplegia persisted. In light of his persistent hypoesthesia and paraplegic gait with developing neuropathic pain, he was readmitted, and an MRI of his lumbar spine revealed multilevel degenerative disease and tethered spinal cord syndrome with compression of the medulla at the L2–L3 level. The patient underwent central flavectomy with bilateral foraminotomy at the L2–L3 level, and the medulla was decompressed. Postoperatively, his neurological symptoms were significantly improved, and he was discharged from hospital on the third day after admission. In support of our case, we systematically reviewed the recent literature and analyzed cases published between 1949 and May 2022, including clinical features, mechanisms of infection, predisposing factors, radiological investigations, microbial etiologies, therapies and their duration, follow-ups, and outcomes. Initial clinical presentation can be misleading, and the diagnosis can be challenging, because this condition is rare and coexists with other spinal diseases. Hence, a high index of suspicion for making an accurate diagnosis and timely intervention is required to preclude mortality and unfavorable outcomes. Our case is a clear example thereof. Long-term follow-up is also essential to monitor for abscess recurrences.

## 1. Introduction

Intramedullary spinal cord abscess (ISCA) is a rare infectious pathology of the central nervous system. The first case of a patient with ISCA was reported by Hart in 1830 [1,2]. Currently, fewer than 140 ISCA cases have been reported in the literature [3]. The mortality rate and outcome of these infections were improved when antibiotics, advanced neuroimaging modalities, and immediate surgery were accepted into clinical practice. Byrne et al. discovered that 36% of intramedullary abscesses primarily involved the cervical cord, 36% the conus medullaris, and 29% the thoracic cord; in congenital midline defects, lower thoracic and lumbar segments are the common sites [4]. In the pre-antibiotic era, 50% of ISCA cases resulted from hematogenous spreads of infection with an extraspinal focus. However, in a modern-era review of ISCA, most cases are cryptogenic and 8% are linked to hematogenous spread from an extraspinal focus [2,5]. Spinal cord tissue has exceptional resistance to infection. Additionally, the small volume of the cord compared to the brain, and the spinal cord’s small lumen with an acute angle of origin of the spinal arteries have been considered protective factors, minimizing the incidence of ISCA [6]. Therefore, ISCA generally occurs together with underlying systemic conditions such as immunosuppression, diabetes mellitus, structural abnormality, adjacent spinal infection, or intravenous drug abuse. Its presentation can be divided into three types, depending on the time between initial presenting symptoms and time of diagnosis: acute, subacute, and chronic. These types have prognostic significance. The classic clinical presentation includes fever, pain, and an acute onset of neurological deficits, depending on the location. However, the ISCA triad is often absent in subacute or chronic cases [7].

During the pre-antibiotic era, Arzt reported a 90% mortality rate for ISCA cases published between 1830 and 1944 [8]. The mortality rate and prognosis were improved when antibiotics, advanced neuroimaging modalities, and immediate surgery were introduced into clinical practice. Subsequent reviews of published ISCA cases reported mortality down to 24% by 1977, while a more substantial improvement to 4% was described by Kurita et al. for cases published between 1998 and 2007 [9].

There is a bimodal age distribution among adult ISCA patients, most reported cases being diagnosed during the first and third decades of life [10]. The incidence rate is unknown, but the condition is associated with significant morbidity and mortality. Bartel et al. reported a higher incidence in women during their first four decades, whereas men had a constant incidence throughout their lifetime [11]. A high index of suspicion of ISCA is crucial for prompt diagnosis, effective clinical management, and supportive therapy, in order to prevent further neurological deterioration and to secure a favorable outcome [12].

Here, we report a case of a 65-year-old male patient who presented with a progressive spastic gait and lumbar pain and was found to have a thoracic intramedullary abscess with concomitant spinal degenerative disease. He was treated with surgical intervention and subsequent intravenous antibiotics. Following our case report, we also present a systemic review of the 70 adult cases in the current literature published between 1949 and 2022.

## 2. Case Description

**Figure 1:**
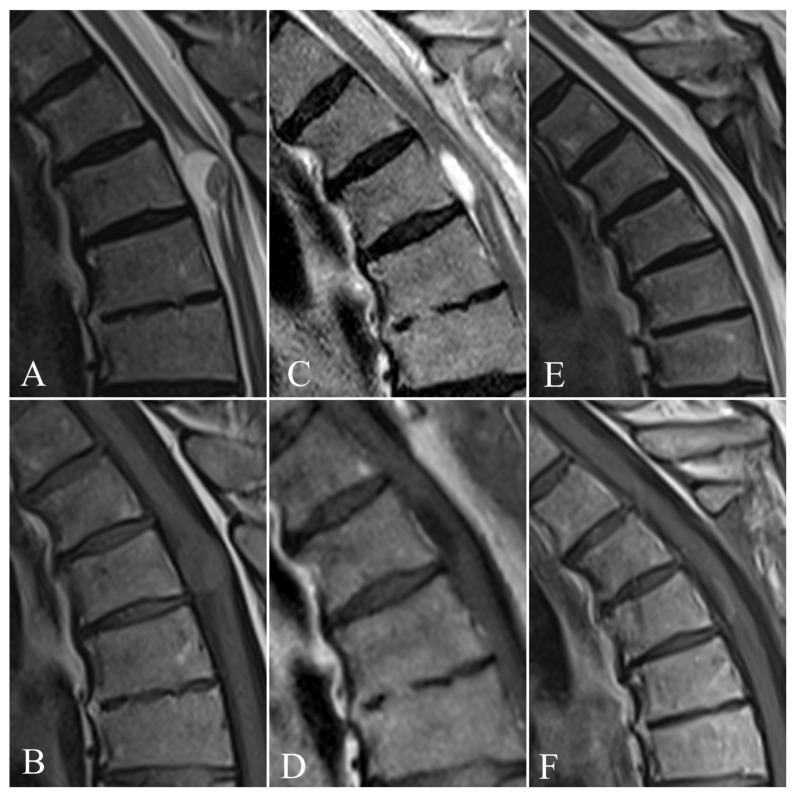
Magnetic resonance imaging (MRI) showing the sagittal scans: (**A**) T2-weighted image and (**B**) T1-weighted images of an expansile intramedullary solid-cystic lesion measuring 16 mm × 11 mm × 10 mm, without contrast enhancement, at the T4 level, with segmental widening of the spinal cord above and below the lesion. The early follow-up MRI shows no pathological mass and postoperative changes on both (**C**) T1-weighted and (**D**) T2-weighted images. The MRI performed 6 months after surgery revealed complete abscess excision in (**E**) T2-weighted and (**F**) T1-weighted sequences.

A 65-year-old male patient was admitted to the Department of Neurosurgery with a progressive spastic gait and lumbar pain radiating to both lower extremities, predominantly in the left leg, without fever, following progressive paraparesis in both lower extremities. He reported difficulty in walking owing to paraparesis of grade 3/5 (Power-McCormick scale) in both lower extremities. During a neurological examination the symptoms progressed, being exacerbated by upright posture. Progressive left foot drop and claudication were also observed. Five years prior, L2/L3 discectomy was performed without any major improvement. The medical history revealed an aortic valve prosthesis implanted fifteen years previously. An MRI with contrast revealed an expansile intramedullary cystic lesion at T4 level measuring 16 mm × 11 mm × 10 mm without any enhancement (Figure 1A,B). Control lumbar MRI was comparable with a study done three years earlier.

After numerous consultations over several months, he was referred to our department by a neurosurgeon, owing to persistent lumbar pain progressing to the thoracic vertebrae and the progressive spastic gait. He had hypesthesia in both lower limbs, the left foot being most affected. There was no nuchal rigidity or other meningeal signs, and he was afebrile. Laboratory tests revealed an elevated CRP level of 70 mg/L. There was no laboratory evidence of immunosuppression. The clinical picture indicated thoracic medulla lesion as the cause of the ailments. The differential diagnosis based on MRI included a neoplastic process; the lesion was presumptively diagnosed as an astrocytoma or ependymoma.

**Figure 2:**
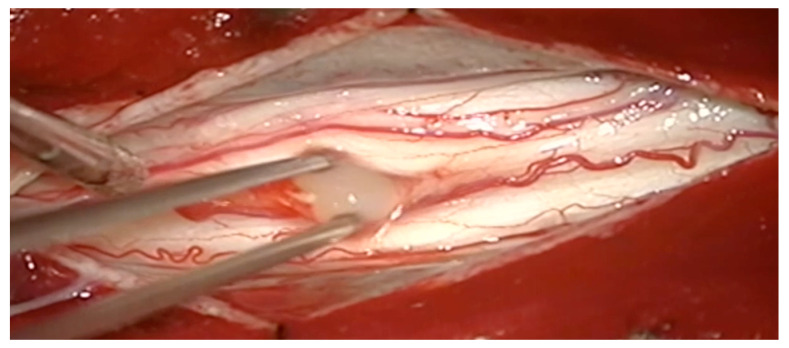
Intraoperative view of lesion showing yellowish pus after myelotomy at T4 level.

A laminectomy with intradural exploration was performed at the T4 level. When the dura was opened, the spinal cord appeared edematous and discolored. There was a yellowish ill-defined abscess capsule endophytic through the ventral side of the spinal cord, which was carefully drained through a midline myelotomy (Figure 2).

The spinal cord became lax after the abscess was drained. We encountered purulent fluid through the midline myelotomy from T3 to T4. The cavity was irrigated with saline, the dura was stitched, and the wound was closed in layers, with drainage placed. A postoperative MRI of the thoracic spine after two days and initial treatment showed complete removal of the intramedullary abscess (Figure 1C,D). A cardiovascular evaluation provided no evidence of infectious endocarditis, nor was there an oral infection. A subsequent pus culture to make a definitive diagnosis revealed Staphylococcus aureus MRSA and Staphylococcus Coagulase-negative. Blood cultures were not performed, due to good clinical state and lack of any infection, especially central nervous system infection. Subsequently, a neurogenic tumor was suspected due to MRI features. Additionally, the definitive diagnosis of degenerative lesion was difficult because no cystic features were indicated on MRI images. The culture was positive for Staphylococcus aureus, and the patient was placed on targeted antibiotics for further management. Therefore, the blood culture was deemed not essential, as it would have been difficult to extrapolate the source of infection and determine whether the infection had been spread hematogenously. We began targeted therapy, and the patient was given three weeks of postoperative intravenous vancomycin regimens.

In control laboratory tests, the infectious parameters decreased significantly, the CRP level falling to 2–3 mg/L after the antibiotic regimen. An MRI of the thoracic spine two days after the operation and initial treatment showed complete removal of the intramedullary abscess (Figure 1B,E). Nevertheless, the patient’s lumbar pain and paresis remained. He was then transferred to another hospital to undergo rehabilitation therapy, and then discharged home.

**Figure 3:**
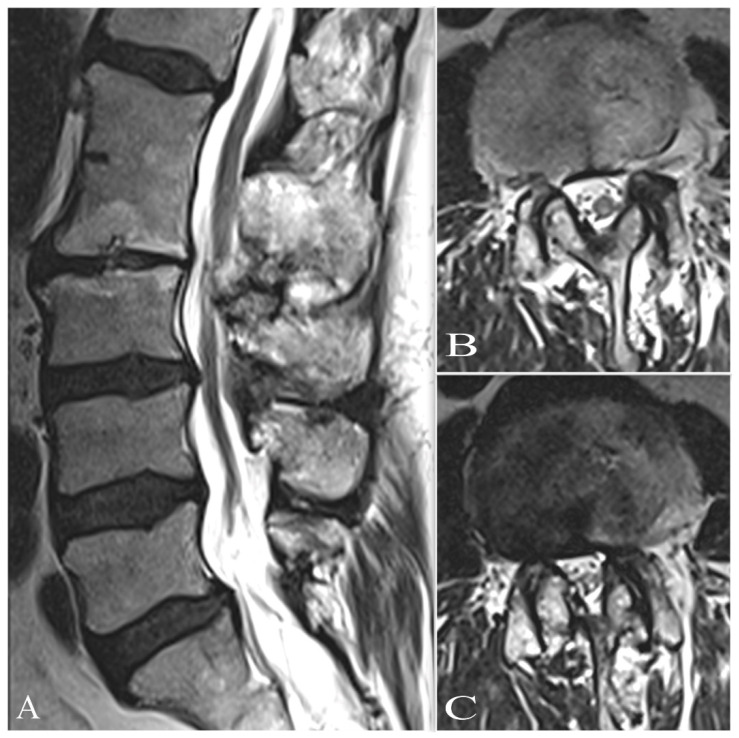
Lumbar multilevel spinal degenerative disease on MRT2-weighted images: (**A**,**B**) sagittal and (**C**) axial view at L2–L3 level; severe stenosis with compression of the conus medullaris.

Control thoracic MRI after six months showed complete removal of the abscess (Figure 1E,F). Progression of paraplegic gait was improved and controlled. However, persistent lumbar pain, hypesthesia, and paraplegic gait were reported, with developing neuropathic pain described as a burning sensation. Another MRI of his lumbar spine confirmed multilevel degenerative disease and tethered spinal cord syndrome, with the medulla compressed by L2–L3 (Figure 3A–C). However, the images were very similar, and a slight progression of L2/L3 stenosis was detected. The elective surgery was scheduled, and three months later, the patient was admitted to the award. Then, a central flavectomy with bilateral foraminotomy was performed at the L2–L3 level, with decompression of the medulla.

Postoperatively, the patient’s neurological symptoms, lumbar pain, and gait were significantly improved, and he was discharged from hospital on the second day.

## 3. Systematic Literature Review

**Figure 4:**
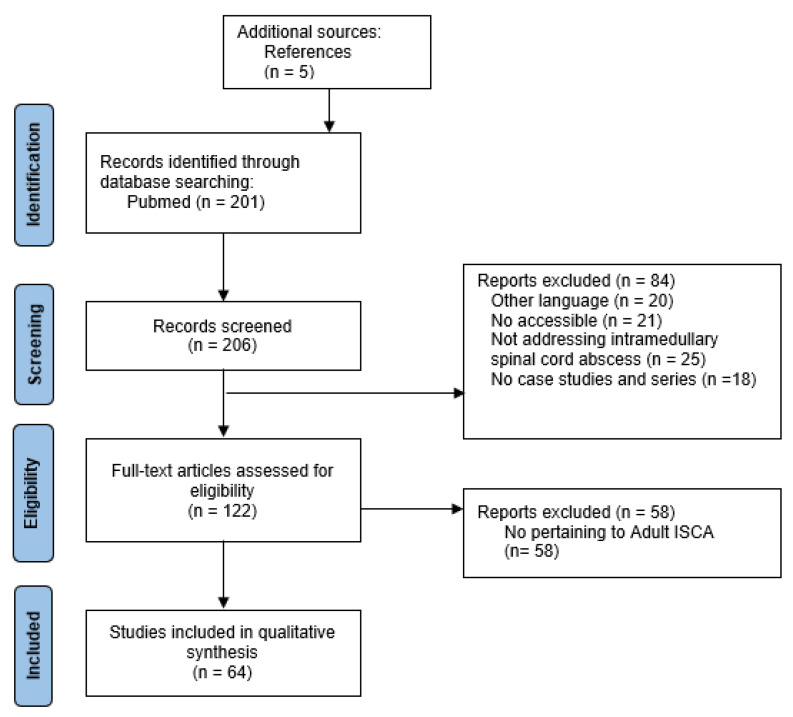
The flow-chart of publications included process.

PubMed was searched for English-language case studies and case series published from January 1949 to February 2022. The key words ((intramedullary), AND (spinal cord)) AND (abscess) were used in the search. Only English language case studies/series pertaining to ISCA in adults were taken into consideration. We also reviewed secondary articles from the references cited in previous research studies (see Section 4: Results, for further information). To facilitate the systematic feature of our work we have added a PRISMA flowchart (see Figure 4).

The demographics and clinical features of patients, mechanisms of infection, predisposing factors, radiological investigations, therapies and their duration, follow-ups, and outcomes were checked for every case. An ISCA case was defined by the following: a culture and a positive/negative Gram staining of a spinal cord aspirate, both revealing a bacterial pathogen; MRI imaging revealing an abscess associated with a specimen from a normally sterile site yielding bacteria; or a positive immunological test for a specific bacterial pathogen. Cases of culture-negative ISCA in which a spinal cord aspirate contained polymorphonuclear leukocytes, and cases where Gram staining and culture of the aspirate were negative, were also included in our study.

The clinical presentation was divided into acute (symptoms lasting less than one week), subacute (symptoms lasting 1–6 weeks), and chronic (symptoms lasting more than six weeks). The mechanism of infection was typically: (1) hematogenous spread of an extraspinal infection; (2) contiguous spread of an adjacent infection; (3) direct inoculation (i.e., penetrating trauma, post-neurosurgery); or (4) cryptogenic (infection source unknown). Outcomes were graded on the basis of ambulatory status, since all patients had motor symptoms in both the upper and lower extremities. The outcomes were grouped as follows: Complete neurological recovery, Residual neurological deficits, Persistent neurological deficits, and Death.

## 4. Results

We found a total of two hundred and six (including five from references) papers on ISCA in literature published from January 1949 to May 2022. We included 122 articles that were full-text papers in English presenting a case study or case series. We identified 137 ISCA cases from 64 papers. Seventy (50.03%) adult cases and 64 (45.99%) pediatric cases were abstracted from the database. Of the 70 cases, 55 (78.5%) were males and 15 (21.4%) females. The median age at presentation was 52 (IQR: 38.5–66.5) years.

### 4.1. Clinical Manifestation and Onset

**Figure 5:**
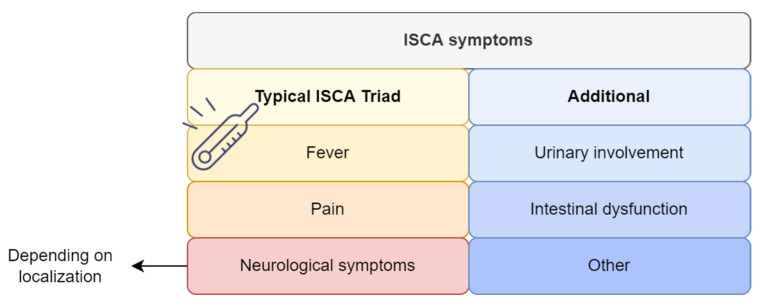
The typical clinical features of intramedullary spinal cord abscesses. Legend: ISCA—intramedullary spinal cord abscess.

The clinical features varied, and depended on the abscess’s location. Symptoms upon admission were fever, back pain, neurological deficits including motor and sensory disturbances, and urinary incontinence, amongst others, often originating from concomitant diseases such as meningitis, encephalitis, and other systemic infections (Figure 5).

Fever and neurological deficits were the most common symptoms, along with pain, and urinary incontinence was present initially in 25 cases (35.7%).

The typical clinical features are summarized in Figure 4. The patients were divided into three clinical groups depending on their clinical presentation and its duration from the onset of symptoms until hospital admission: acute (less than a week) in twenty-six (37%) patients, subacute (1–6 weeks) in twenty (28.5%), chronic (more than six weeks) in fourteen (20%), and not mentioned in six (8.5%). The most common complaint at the time of presentation was motor impairment, found in 69 cases (98.5%), followed by sensory loss in 61 cases (87.1%), pain in 39 (55.7%), and urinary involvement in 35 (50%).

Concerning concomitant diseases, the following comorbidities were reported (see Table S3): diabetes and concomitant systemic infection (n = 5), urinary tract infection (n = 4), chronic kidney diseases (n = 1), pulmonary diseases (n = 2), tuberculosis (n = 2), chronic sinusitis (n = 1), oral infection (n = 2), infective endocarditis (n = 2), sepsis and septic emboli (n = 3), systemic lupus erythematosus (n = 1), sickle cell disease (n = 1), pulmonary arteriovenous fistula (n = 1), spinal dural arteriovenous fistula (n = 1), vertebral infection (osteomyelitis, discitis and spondylodiscitis, arthritis) (n = 4), spinal anatomical abnormalities (n = 3), CNS histoplasmosis (n = 1), disseminated coccidioidomycosis (n = 2), ulcerative colitis (n = 1), and heroin/alcohol addiction (n = 5).

### 4.2. Microbiology

A bacterial pathogen was found in 64% of cases (Table 1). Four infections were polymicrobial [13,14,15,16]. The diagnosis was confirmed by positive culture of a spinal cord aspirate and biopsy in 14 (20%) cases, with additional isolation of Escherichia coli from urine culture (case 38). In four cases [4,17,18,19], the diagnosis was based on the identification of Gram-negative bacilli on a Gram stain of an aspirate. For 30 other patients, the diagnosis was based on a CSF culture, and *Escherichia coli* and *Enterococcus fecalis* were isolated from the patient’s urine culture in three cases [17,20,21] and a blood culture in three [22,23,24], needle aspiration and abscess culture [6,14,25,26], and histopathology [1,20,27]. The remaining four cases [28,29,30,31] were classified as culture-negative ISCA. Two cases [25,32] had previously received antimicrobial therapy. Two others were diagnosed via blood culture [21,24], and the others were identified via urine culture [33] and histopathology [34]. There were elevated white blood cells and leucocytes in the remaining 11 (15.71%), and the diagnostic tool was not mentioned in four.

The most common causative organisms isolated in culture were *Streptococcus* spp. in 14 cases (20%) followed by *Staphylococcus* spp. in 10 (14.28%). The other organisms identified were *K. pneumoniae*, *C. albicans*, *M. tuberculosis*, *Nocardia* spp., *Actinomyces*, *Aspergillus*, *Bacteroid* spp., *Brucella* spp., *MRSA*, *E. coli*, *Pseudomonas* spp., *L. monocytogenes*, *H. capsulatum*, *Hemophilus* spp., and *E. fecalis*. The culture of pus in the present case revealed *Staphylococcus aureus.*

### 4.3. Mechanism of Infection and Predisposing Factors

Thirty-two patients (45.7%) had cryptogenic sources, including our case. Seven (10%) resulted from a spread of infection through contagious lesions, such as spinal anesthesia [43], dermal sinus tracts [38], epidural abscesses [48], epidural anesthesia [40], intrathecal morphine pump [49], spondylodiscitis [50], vertebral osteomyelitis [47], vertebral discitis, and osteomyelitis [51]. Patients with anatomical abnormalities of the spinal cord and/or vertebral column were found in four cases (5.7%). Three (4.2%) had congenital midline neuroectodermal defects (spinal dysraphism). One patient (1.4%) with congenital midline defects had related dorsal midline skin lesions, namely a dermal sinus tract opening with prior discharge from a sinus tract, accompanied by meningitis. Four other cases (5.7%) had direct penetrating trauma to the spinal cord through stab wounds, by wooden foreign bodies and surgical excision of spinal epidermoid and ependymoma [13,15]. In two other cases, spinal abscesses were secondary to postoperative complications following resection of a spinal cord dermoid cyst [38] and spinal cord ependymoma [25]. Case [29] had a cervical spinal stenosis, extrinsically compressing the spinal cord. Twenty-two cases (31.4%) had extraspinal hematogenous spreads: urinary tract infection [6,21,27], pyelonephritis [19], bronchopneumonia [52], bronchiectasis [53], tuberculosis and SLE [26,39], brucellosis (32), oral infections and dental procedures [54,55], chronic kidney diseases [17], infective endocarditis [22,23,30,51], diabetes mellitus with systemic effects [21,22,42,45], disseminated coccidioidomycosis [28], histoplasmosis [56], and neurotuberculosis [57]. Three patients (4.2%) had apparent sepsis originating from either *E. coli* [19], *S. aureus* [48], or Gram-negative bacilli [27]. One case had multiple pulmonary arteriovenous malformations [16], and another [34] had SDAVF, which are both known to be risk factors for the development of pyogenic brain abscesses.

### 4.4. Neuroimaging Features

Neuroimaging studies such as plain radiography, myelography, computed tomography scans (CT) and MRI were carried out for all but five of the patients involved in the reports. MRI was performed with contrast material in 50 cases. The findings were ring-enhancing margins, segmental widening and swelling of the spinal cord, partial or total obstruction of the cerebrospinal fluid, and tethered spinal cord in some cases [25,58,59]. The radiological features are reported in Table 2.

### 4.5. Management and Clinical Outcomes

Thirteen patients (18.6%) required urgent treatment and 11 (15.7%) were treated within a day to three weeks from onset. One (1.4%) was treated within a year; there were no data on duration for the rest of the cases. Surgery, including drainage procedures (laminectomy and myelotomy in 41 cases (58.5%) and stereotactic needle aspiration in one), was performed in 44 cases (62.8%). A second open-drainage procedure was required in case [18] following deterioration of neurological functions. Additional surgical procedures included excision of the abscess cavity (EAC) in four cases (5.7%), excision of a sinus tract (EST) in two (2.8%), excision of an epidermoid cyst (EEC) in one (1.4%), marsupialization of the abscess cavity in one (1.4%), and a biopsy in two (2.8%). Surgical intervention was not mentioned in 18 cases (25.7%). Antimicrobial therapy was administered for durations of nearly two weeks to nine months in 59 (84.2%) cases; for eight patients (11.4%) its use was not mentioned, and only one patient (1.4%) continued therapy for one year.

The clinical outcomes were recorded after follow-ups ranging from nearly two weeks to one year for all but one patient. We categorized the other cases into four groups on the basis of their prognoses: recovery in 20% of cases (Table 1), residual ND in 12 (17.1%) (Table 3), persistent ND in 28 (40%) (Table 4), and death in 10 (14.2%) (Table 5). All but two (2.8%) in the recovery group had received antimicrobial therapy, 11 (15.7%) underwent surgery, and the time frame between onset and first treatment (surgery or antibiotics) was urgent (IQR: urgent—six days). Among the 60 patients (85.7%) who survived, residual neurological deficits such as paraparesis and paraplegia persisted in 40 (57.1%). Among these, 59 (84.2%) received antibiotics and 44 (62.8%) underwent surgery. The median duration from onset to first treatment for the persistent group was 1.5 days (IQR: urgent-4.74), while for the residual group it was two days (IQR: 0.5—4.25). However, there were too few data in the death group about time from symptoms to treatment; information was only available in one case (10%). Ten patients (14.2%) died postoperatively: as a result of septic embolus with concomitant pyelonephritis in [19], UTI [6,33], chronic alcoholism and bronchopneumonia in case [52], disseminated coccidioidomycosis [28], multiple cerebral abscesses complicated by progressive hydrocephalus [53], abscess rupture inducing meningitis and brain abscess [53], Listeria meningoencephalitis [12], central nervous system nocardiosis involving the bilateral hemisphere, cerebellum, and upper cervical spinal cord due to diabetes mellitus (despite a two-stage operation) [20], cardiac arrest [51], intracranial extension of her spinal aspergillosis resulting in rapid progression of ventriculitis and cerebral vasculitis with diffuse vascular occlusion and widespread cerebral infarction [51], and refractory septic shock [60]. There were no significant differences in the time until treatment among the recovery, persistent, residual and death prognosis groups (*p* = 0.613).

## 5. Discussion

**Figure 6:**
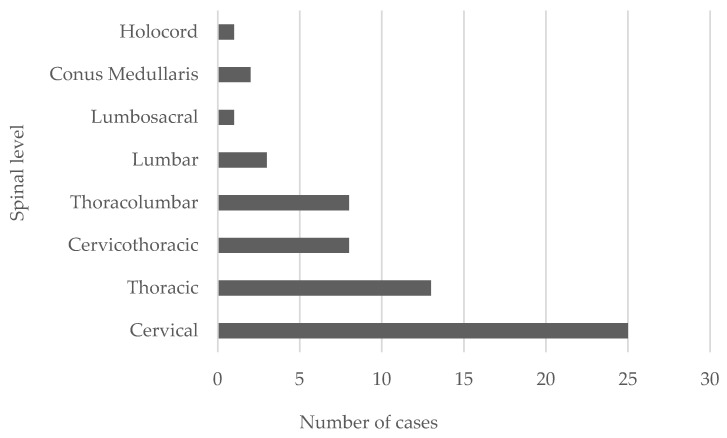
The typical localization of spinal cord abscess in 61 patients with intramedullary spinal cord abscess.

ISCA is rare neurological entity in spinal cord infection, fewer than 140 cases having been reported since its first description by Hart in 1830. These infections traditionally correlate with high morbidity and mortality [37]. Furthermore, they can be located throughout the spinal cord, and multiple abscesses occurred in 26% of cases [73]. Byrne et al. discovered that 36% of intramedullary abscesses primarily involved the cervical cord, 36% the conus, and 29% the thoracic cord, whereas lower thoracic and lumbar segments are the common sites in congenital midline defects [4]. In previous studies, intramedullary spinal cord abscesses mostly involved the cervical and upper segments of the thoracic cord [74]. In our review, twenty-five abscesses (35.71%) primarily involved the cervical cord, nineteen (27.14%) the thoracic cord, three (4.2%) the lumbar cord, eight (11.42%) cervical to thoracic, eight (11.42) thoracic to lumbar, one (1.42%) lumbar to sacral and holocord, and two (2.85%) the conus; four cases had multiple abscesses (see Figure 6). In the cases between 1944 and 1975, the average spinal cord abscess length was six levels, and the abscesses grew preferentially along fiber tracts longitudinally [75]. The reduction in the size and number of spinal cord abscesses in recent cases can be attributed to improved early detection, surgical intervention, and antibiotics (Byrne). Two-thirds occur during the fourth decade of life, predominantly in males (M:F ratio 5:3) [73]. A bimodal age distribution among adult patients has been described; most patients were diagnosed with ISCA during the first and third decades of life. The incidence rate in women was higher during the first four decades, whereas in men it was constant throughout their lifetime [11,24,63].

### 5.1. Etiology and Pathogenesis

According to the available literature, spinal abscesses begin in the central gray matter and extend peripherally into the white matter. The suppurative infiltrate causes inflammation with a predominance of polymorphonuclear cells and liquefactive necrosis-producing enzymes. The necrotic area is encapsuled by fibroblasts and the abscess then extends caudad and cephalad, separating the fiber tracts without compression until the later stages of the disease. Fibrous proliferation and gliosis, both rostral and caudal to the abscess cavity and surrounding the adjacent areas of necrosis, are found on histopathological examination and could cause the signal changes on the MRI [65]. Polymorphonuclear leukocytes and lymphocytes often thicken and infiltrate the meninges, with frequent venous thromboses transversing the areas of abscess [37]. ISCA is a suppurative infection with abscesses that develop similarly to pyogenic brain abscesses, normally concomitant with other pathologies. Normal spinal cord tissue has an exceptional resistance to infection [61]. ISCA generally occurs together with underlying systemic conditions such as immunosuppression, diabetes mellitus, or intravenous drug abuse [76].

Several risk factors (especially for cervical spinal abscess) including dermoid cysts, ependymoma, meningomyelocele, osteomyelitis, pre-existing spinal pathologies including spinal tumors, dural arteriovenous malformations or fistulas, previous surgeries and iatrogenic or stab wounds, penetrating or iatrogenic injuries to the spine, intravenous drug use and alcoholism, genitourinary infections, infective endocarditis, pulmonary disease, septic embolism, and bacterial meningitis can precipitate intramedullary infections [17,22,24,44,46,51,61,65,77,78,79]. Other predisposing factors for intradural infection and ISCA in children are congenital midline defects such as anatomical abnormalities of the spinal cord or congenital dermal sinus [7]. According to our literature search and review, we can categorize these conditions into four groups: bacterial and fungal infections, penetrating trauma to the spinal cord, congenital dural sinuses, and chronic tuberculosis. Intramedullary abscess formations have rarely been associated with acute bacterial meningitis [61]. Bartel et al. found the primary focus of infection in 7.5% of cases. An immunocompromised state associated with diabetes, HIV infection, prolonged steroid therapy, and drug addiction also appear to be important [11]. However, our patient was neither immunocompromised nor apparently subject to any of those predisposing conditions.

### 5.2. Classification

#### 5.2.1. Mechanism of Infection and Predisposing Factors

Classifying ISCA according to infection mechanisms enables us to predict the organism(s) most likely to be causal. The causative factors of ISCA development can be grouped as follows: hematogenous spread (i.e., extraspinal focus of infection), contiguous spread (i.e., adjacent focus of infection), and direct inoculation (e.g., via penetrating trauma, neurosurgery, or a cryptogenic mechanism). Hematogenous spread appears to be most common; we thought this was the case with our patient, but we could not identify the source of the infection. The patient’s prior surgery and aortic prosthetic valve were assumed to be the only important predisposing factors; therefore, the infection mechanism remains cryptogenic [2,80,81].

During the pre-antibiotic era, 50% of cases were due to hematogenous spreads of infection with an extraspinal focus; nearly 20% had an underlying infectious lung disease. Other main sites of infection included soft-tissue infections and infective endocarditis [2]. In a modern-era review of ISCA, Chan and Gold et al. found that 44% of cases were linked to anatomical spinal cord or vertebral column abnormalities. Twenty-four percent (24%) of cases resulted from a contiguous infection spread through sinus tract openings, 8% from hematogenous spread with an extraspinal focus, and 64% were cryptogenic. A bacterial pathogen was identified in 64% of cases, while the remaining cases were classified as culture-negative ISCA [2,37].

Organisms typically found in cryptogenic cases were Listeria monocytogenes, Streptococci viridans, Hemophilus spp., Enterobacteriaceae, anaerobes, anaerobic streptococci, and oral anaerobic Gram-negative bacilli. Therefore, careful examination of the skin of a patient exhibiting neurological symptoms suggestive of a spinal cord syndrome can provide an early clue to the diagnosis of ISCA. In these cases, the causative organisms that reflect skin-colonizing species such as *S. epidermides*, *S. aureus*, Enterobacteriaceae, and anaerobes including Bacteroides fragilis are related to ISCA cases in the dermal sinus tract. Additionally, in the lumbar region, pathogens commonly include enteric Gram-negative rods and anaerobes as well as Staphylococcus species [37].

Although ISCA can result from hematogenous spread from an extraspinal source, Hoche demonstrated that the introduction of microorganisms into the CSF of animals did not cause ISCA unless thrombi were also introduced. This led to the discovery that ISCA is rare without pre-existing spinal cord abnormalities. The major sources of hematogenous spread (42%) are from the urogenital tract (e.g., vulvovaginitis, urinary tract infection, pyelonephritis, or perinephric abscess), followed by pneumonia, endocarditis, middle ear infection, and sagittal sinus thrombosis. Schistosomiasis and brucellosis are also possible hematogenous sources. Direct spread of the microorganism from adjacent structures is not as frequent, but if this occurs it is typically after spinal procedures, the administration of epidural anesthesia, or vertebral osteomyelitis [2,76,80].

ISCA during the pre-antibiotic and modern eras resulted in similar proportions of contagious cases (38% vs. 24%). Only around 10% of infections in the pre-antibiotic era were found in congenital dermal sinuses [2]. In contrast, several infections resulted from the direct extension of local, deep-seated infections [82].

Most recent ISCA cases are cryptogenic, probably arising from transient bacteremia of mucosal surfaces or clinically unknown extraspinal sites of infection. Several lines of evidence support the hypothesis that this transient bacteremia can seed areas of subclinical spinal cord injury or microinfarction. In the present review, nearly 20% of cases of cryptogenic ISCA were in patients with structural abnormalities in their spinal cord and/or vertebral bodies (spinal cord ependymoma, previous resection of a spinal cord lipoma, or spinal stenosis). Furthermore, cryptogenic cases were mainly located in the cervical and upper thoracic segments of the spinal cord [2]. Additionally, around 25% of cases were from contiguous spread of infection from dermal sinus tracts. Predisposing factors in pediatric ISCA include congenital midline defects and anatomical abnormalities of the spinal cord or vertebral column [74]. This shift in pathogenesis is likely to be related to the wide availability of effective antimicrobial agents to treat the primary sites of infection. Moreover, advances in non-invasive imaging could recognize the primary sites of infection earlier.

Sterile culture was the most common finding in earlier reports. This also applies in our review, especially in chronic cases. In one chronic case the culture was probably sterile because of prior administration of antibiotics. Although 30% of spinal abscesses are microbiologically sterile, a diverse range of organisms have been identified amongst the remaining 70% such as Staphylococcus (the most frequently reported cause at 25%), Streptococcus, Escherichia coli, Proteus, Listeria, Bacteroides, Pseudomonas, Brucella, Hemophilus, Histoplasma, Actinomyces, and Mycobacterium [83]. Anaerobes are uncommon, but many types of microorganisms have often been found in spinal abscesses in patients with ISCA [79]. Common causes of intramedullary abscesses include Gram-positive cocci, especially those native to oral or skin flora [13,14,20,27,30,37,56,64,84]. Implications for the selection of an empirical antimicrobial regimen in this population have arisen because opportunistic pathogens such as *Fusarium* spp. or *Aspergillus* spp. have been reported as causing intramedullary abscesses in immunocompromised patients. This further emphasizes the importance of obtaining culture data for direct therapy. When broad empirical coverage is started, clinicians should consider not only the wide array of pathogens that can cause intramedullary abscesses but also the patient’s risk for tuberculosis and fungal infections, and their immune status [26,85]. The outcomes of these infections have apparently improved since antibiotics were introduced into clinical practice. The first review of ISCA cases 1830 and 1994 reported a mortality rate of 90%. A review between 1994 and 1997 reported a mortality rate of only 8%. Kurita et al. described a case of cervical ISCA treated with antibiotics alone, but whether this suffices is a matter of debate [2,8,9]. Despite the improvement of survival rates since the pre-antibiotic era, neurological deficits persist in most patients who survive. In our patient, the mechanism of infection appeared to be hematogenous spread, though we could not identify the origin of infection. We assume that the prior neurosurgical procedure and aortic valve implantation were the important predisposing factors, which could have caused bacteria to seed to the intramedullary space of the thoracic spinal cord. Thus, the mechanism of infection remains cryptogenic in this case. His wound culture from the ISCA was positive for *S. aureus*, which is part of the oral flora and is consistent with our proposed etiology.

#### 5.2.2. Onset and Clinical Presentation

According to Foley, the time between initial presenting symptoms and time of diagnosis has prognostic indications. The presentation of ISCA can be divided into three clinical groups: acute, subacute, and chronic [6]. Acute symptoms last less than a week, and patients are more likely to have a fever and leucocytosis. In the subacute and chronic groups, the symptoms persist, respectively, from 1–6 weeks and for more than six weeks. These two groups are unlikely to present with fever and leucocytosis.

Acute spinal cord abscesses often follow the same clinical course as transverse myelitis, whereas chronic abscesses resemble an expanding spinal cord tumor. Our patient had an acute onset with neurological worsening. Menezes et al. observed that acute presentations had worse outcomes; patients with symptoms for less than four days had a 90% mortality rate, while those with symptoms lasting more than a week had a 60% mortality rate [86].

Motor and sensory deficits (68%), pain (60%), bladder dysfunction (56%), fever (less than 50%), meningmus (12%), and brainstem dysfunctions (4%) were the most common presenting complaints [9,87]. The triad of ISCA is fever, pain and neurological deficits, but not all patients show all three; the ISCA triad is commonly absent in subacute or chronic cases [7]. Signs and symptoms suggesting a structural lesion of the spinal cord, as opposed to features that suggest infection, are most common. Spinal epidural abscess presentations show the same pattern, so epidural abscesses should always be included in the differential diagnosis. Without concomitant vertebral osteomyelitis, primary spinal epidural abscesses are rare. This correlation could assist in a differential diagnosis of subdural and epidural spinal abscesses [2,88]. As was observed in our patient, the presentation of intramedullary abscesses is frequently marked by progressive dorsal pain followed by neurological deficits. Our patient’s onset was acute, and four of the main clinical signs were present. The clinical presentation can be insidious and can imitate a spinal tumor or other chronic myelopathy-inducing condition. The multifocality of abscesses causes further complications within an already complex clinical picture [89]. Finally, meningitis can recur as a result of abscesses rupturing into the subarachnoid space [67,79]. For the purpose of management, it is crucial to differentiate ISCA from other tumorous pathologies so that adequate medical and surgical treatment can be initiated promptly.

### 5.3. Evaluation and Management

Laboratory data can help in making a definitive diagnosis when there is clinical suspicion of a spinal cord abscess. Clinical findings of ISCA are nonspecific and do not lead to a final diagnosis. Leucocytosis and elevated inflammatory markers (e.g., increased platelets, C-reactive protein, and/or erythrocyte sedimentation rate) are common. There is abnormal CSF in 78% of patients, and tests can indicate elevated leucocytosis, decreased glucose, increased protein, and/or a positive Gram stain. Nonetheless, CSF test results can be normal in spinal abscess cases [76].

### 5.4. Neuroimaging Features

The main route to diagnosing patients with spinal abscesses has traditionally been radiological. Conventional radiographs and myelography were the only imaging modalities used for diagnosing spinal abscesses, and the only diagnostic approaches mentioned in literature, until 1980. This was prior to the widespread availability of computed tomography (CT) scans and magnetic resonance imaging (MRI) [90].

A spinal intramedullary ring-enhancing mass is a nonspecific imaging feature in various non-inflammatory benign and neoplastic processes, but rarely in ISCA [36]. Because ISCA is very rare, MRI features have only been described in single case reports and seem to resemble changes in brain abscesses [91]. Extended hyperintensity of the spinal cord on T2-weighted images with a circular enhancement on T1-weighted images were included after intravenous application of gadolinium chelates [36]. Edema has a range of severities and the grade of contrast enhancement depends on the lesion stage. MRI is mostly performed in a later capsular stage, since clinical manifestations are often nonspecific and only 40–50% of patients are febrile upon examination [36]. In our study, a hypointense abscess capsule was seen on MRI T2-weighted images, which we assumed to be caused by the susceptibility effects of free radicals and extended edema of the thoracic cord.

Features that suggest a structural lesion rather than signs of infection are more often found in ISCA presentation. MRI with contrast is the gold standard for accurate identification of the location and extent of an abscess and for identifying any predisposing structural abnormalities of the spinal cord [80]. The MRI features of ISCA include enlargement of the spinal cord with increased signal intensity on T2-weighted images and marginal enhancement with central low signal intensity on T1-weighted images with gadolinium administration. Several non-inflammatory benign and neoplastic processes have a similar ring-enhancing appearance, so this is a nonspecific imaging finding for abscesses. Enhancement can occasionally show extension to an adjacent dura or epidural space [2,7,36,87,88,91,92]. Spinal cord tumors (necrotic glioma, metastases), resolving hematoma, infarction, granulomatous disease, and demyelinating disease (multiple sclerosis) can be included in the radiographic differential diagnosis of a ring-enhancing mass [36,91]. Hyperintensity on T2-weighted images tends to resolve markedly as infections are suppressed by treatment [12,92]. An ISCA that could require immediate surgery should always be considered, although MRI features can resemble those of a tumor, which would not necessarily be considered for immediate resection.

In recent years, diffusion-weighted imaging (DWI) has been added to the list of diagnostic imaging techniques and has been recommended as a more sensitive and specific method for differential diagnosis of abscesses and cystic or necrotic tumors, since the latter present with low signal intensity and show increased values of ADC [40,93,94]. Pus in an abscess cavity is a thick mucoid fluid containing inflammatory cells, bacteria, necrotic tissue and high viscosity proteinaceous exudates. Water mobility and the microscopic diffusional motion of water molecules are heavily impeded, leading to a decreased ADC in the abscess center [40,95]. Necrotic tumor-tissue debris, fewer inflammatory cells than in an abscess, and clearer serous fluid than pus are the usual contents of the cystic and necrotic cavities of tumors. However, an abscess cavity can have variable hyperintense signal intensity on DWI and low ADC values because of a difference in the concentration of inflammatory cells and particularly in abscess fluid viscosity [36]. This reflects decreased diffusion, which can help to distinguish ISCA from cystic spinal cord tumors. DWI signal intensity is higher in abscess cavities than in normal brain parenchyma, indicating restricted diffusion, whereas the signal intensity is generally hypointense in tumor cysts and necrosis, indicating more isotropic diffusion, as in CSF [91]. Previously described precipitation or drop signs characterized by an accumulation of pus at the conus medullaris could be more specific for spinal abscesses [96]. In clinically atypical acute myelopathies, a DWI can also help to distinguish spinal cord infarction from inflammatory myelopathies such as multiple sclerosis, ADEM, neuromyelitis optica, and parainfectious myelopathy [93]. The viscosity depends on the age of the abscess, the etiological organism, and the host’s immune response [36].

### 5.5. Management and Therapy

Prognosis is affected by the extension and aggressiveness of the infective agent and the treatment applied. There is no significant difference between surgically and non-surgically treated patients in the frequency of residual neurological injury. Antibiotic treatment alone can be as effective as surgery plus antibiotic therapy [22].

Prompt diagnosis and intervention are essential for a prognosis and to prevent morbidity and mortality [9,87]. Ages under 25 correlates with good or full recovery, while poor outcomes are more likely after an acute presentation of spinal cord abscess [5,87]. As in the treatment of pyogenic brain abscesses, which are often polymicrobial, a combination of antimicrobial and surgical therapies is recommended for treating ISCA [21].

An interdisciplinary approach involving specialists in neurosurgery, neuroradiology, and infectious diseases is recommended for managing ISCA. Effective prompt surgical evacuation through limited laminectomy and myelotomy, with copious irrigation with normal saline followed by antibiotics according to culture and sensitivity, is the treatment of choice since significant tissue injury can result from the space-occupying nature of lesions. This can limit the extent of neurological injury [16,97].

An open surgical approach (laminectomy and myelotomy) or stereotactic needle aspiration of the lesions under CT guidance can achieve diagnostic and therapeutic drainage of the abscess [2]. There are reported cases of non-surgically treated patients in whom the abscess did not extend more than 2.2 vertebral bodies [7].

Bartels et al. reported that 63% of cases were treated with surgery and 37% without. In the surgical group, 13.6% died, 75% of whom had not received antibiotics. Among those who survived with surgery, 22% recovered completely, 56% improved neurologically, 7% remained unchanged, and one deteriorated. The non-surgical group had 100% mortality. Only 3% of the non-surgically managed patients were treated with antibiotics because 82% of those cases occurred during the pre-antibiotic era. The mortality rate decreased significantly from 90% in the pre-antibiotic era to 8% more recently, which can be attributed to both the development of powerful antibiotics and earlier diagnosis through modern imaging modalities. However, neurological deficits remained in more than 70% of surviving patients [11,24].

The presumed mechanism of infection and the results of Gram staining of aspirate should be the basis for empirical antimicrobial therapy. Since a wide range of microbes can be isolated from an ISCA culture, early treatment with antibiotic combinations effective against *Staphylococcus* spp., *Streptococcus* spp., enteric Gram-negative bacilli, and anaerobes should be used until the causative organisms in each individual case are identified. Chan and Gold recommend including ampicillin in the initial treatment regime for all cases of cryptogenic ISCA to combat L. monocytogenes [2]. Although the optimal duration of antimicrobial therapy is still debatable, a minimum of 4–6 weeks of parenteral antibiotic treatment is recommended, while some authors have proposed the use of intravenous antibiotic therapy for more than nine weeks. Two to three months of additional oral antimicrobial therapy is worth considering [76,97,98,99,100]. Park et al. suggest adjusting the duration of treatment according to the level of ISCA resorption, as indicated by MRI. Using steroids as an adjuvant therapy to decrease edema was recommended in a previous study [101]. Our patient underwent surgery because of his afebrile back pain and persistent neurological deficits. We performed a laminectomy, a myelotomy, and drainage of pus followed by irrigation with saline. The culture yielded *S. aureus*, but his paraplegia did not improve. Accordingly, the patient underwent rehabilitation but his gait impairment persisted and the pain symptoms exacerbated. Another control MRI comparable with previous control MRIs, revealed slight progression of L2–L3 stenosis.

In the view of patient’s progressive stenosis and lumbar pain, an elective surgery was planned. We avoid a major intervention with selective minimal invasive L2/L3 surgery. Postoperatively, patient experienced a dramatic improvement in pain and neurological symptoms. These findings were accidental as the degenerative changes were present before paraplegic gait, with concomitant tethered spinal cord syndrome (medulla to the L3 level) and conus medullaris compression by the L2–L3 stenosis.

Thorough history taking and clinical investigation is fundamental to establish the and differentiate the concomitant diseases that might cause similar clinical symptoms. This can be misleading in establishing definitive diagnosis and tailoring the best therapeutic strategies in cases with similar clinical presentation.

### 5.6. Outcome and Follow-Up

Evaluation of the patient’s response to therapy should be based on follow-up neurological assessments and the results of serial CT or MRI scans to document the resolution of the lesions. Regular follow-up via clinical examinations and MRI during the first year following surgery is highly recommended because intramedullary spinal abscesses recur in 25% of cases. Kurita et al. reported a successfully treated case with the use of antibiotics alone, while Gupta et al. reported a bad outcome from medical treatment alone and emphasized the need for early surgical drainage. There were poor outcomes for ISCA in the pre-antibiotic era, but favorable outcomes can be reached through prompt diagnosis, early and adequate surgical intervention, and antibiotics [1,9].

We were delayed in treating our ISCA case accurately because of the initial misdiagnosis and the patient’s severe paraplegia, which showed no neurological recovery after first surgery. This initial failure to improve was probably caused by a neurological insult incurred from infarction of the cervico-dorsal cord and the delay in surgical treatment. After 11 months the patient underwent the second surgery, after which his neurological deficits improved, and he was able to walk with support after three days.

In general, a good prognosis can be achieved by early drainage and antibiotic infusion [1]. The only significant factors influencing the outcome for ISCA patients are the administration of antibiotics and the time between the onset of symptoms and surgery. Simon et al. reported the deaths of 20% of ISCA patients, residual neurological deficits in 60%, and a 20% recovery rate without sequelae. Patients who did not develop neurological sequelae continued with surgical drainage at a median time from surgery of 1.5 days (range 1–8 days) after hospitalization. Patients who developed neurological sequelae continued with surgical drainage for a median time from surgery of four days (range 1–78 days) after hospitalization. The median time from the onset of symptoms to surgery for those without neurological sequelae was seven days; it was 22 days (range 2–1088 days) for those who developed neurological sequelae. Moreover, a patient’s outcome can be related to the pace of onset of symptoms. Patients developing symptoms in under four days have a mortality rate of 90%, while those whose symptoms last more than seven days have a mortality rate of 67% [76]. Recently, Kurita et al. found no significant difference between surgically and non-surgically treated patients in the frequency of neurological sequelae, but the number of cases in their study was small and the surgically treated group had significantly more extensive abscesses. Thus, patients should receive an appropriate treatment strategy according to their clinical state. A number of patients still experience marked functional neurological impairment, including paraplegia, because of recurrent or chronic abscess formation or spinal cord infarction, despite better prognoses today thanks to advanced surgical techniques and effective antibiotic treatment [1,9]. Our review also revealed that the recovery group received earlier treatment than the persistent, residual, and death groups, though the difference was not significant. Early diagnosis and surgical evacuation, along with broad-spectrum antibiotics, can result in a favorable neurological outcome, as evidenced by our case.

The present case is unique due to its preoperative neuroradiological definitive diagnosis and atypical neuroimaging features, as the findings of MRI were not pertinent for diagnosis of spinal cord abscess. The MRI images did not reveal the characteristic findings of intramedullary spinal cord abscess, including expansion of spinal cord by a ring-enhancing lesion and surrounding edema, which resulted in difficulties in preoperative neuroradiological definitive, and initial diagnosis of neurogenic tumor with abscess. Therefore, we believe that this unusual case will be useful in the diagnostic and patient-specific treatment planning for clinicians and neurosurgeons encountering similar cases of ISCA.

## 6. Conclusions

ISCA is rare and may be associated with underlying pathological conditions that can lead to the formation of a spinal intramedullary abscess. Initial symptoms can be misleading, so a thorough history with precise localization and early diagnosis is valuable. Serious consequences, including risks of severe sequelae and mortality, can result from intramedullary abscesses. A high index of suspicion for proper diagnosis and timely intervention is required to prevent mortality and neurological injury. Contrast-enhanced MRI and novel application of DWI at appropriate levels is the ideal investigation for accurate diagnosis. Any misdiagnosis or delay in adequate treatment can lead to unfavorable outcomes, though the diagnosis is likely to be challenging because the condition is rare. Our case is a clear example thereof. Long-term follow-up is also essential in order to monitor for abscess recurrences.

## Figures and Tables

**Table 1:** Recovery group—summary of the clinical data, patient demographics, clinical manifestations, duration, microbiology, interventions and therapies, and outcomes of contemporary case reports on intramedullary spinal cord abscess (ISCA) in the current literature: systemic review of literature (1949–2022).

No.	Age	Sex	Onset	Location	Infl.	Symptoms		Symptoms to Treatment	Neurosurgical Management	Antibiotics		Pathogen	MOA	Follow-Up	Outcome	Ref.
						Name	Duration			Name	Duration					
1	64	M	Acute	N/D	+	ND (M + S)	N/D	N/D	N/D	Flucytosine Amphotericin B IV	10 d	*C. albicans*	Cryptogenic	1 y	Recovery	[35]
2	27	M	Acute	C5	+	ND (M)	ND	N/D	Stereotactic needle aspiration	Ampicillin IV, Metronidazole IV, Trimethoprim-Sulfamethoxazole IV, Ampicillin PO	3 w	*H. aphrophilus, A. meyeri*	Cryptogenic (Intrapulmonary AVF causing a right-to-left shunt)	N/D	Recovery	[16]
3	80	F	Acute	T8	+	ND (M)	3 d	3 d	N/D	Ceftriaxone, Dexamethasone Metronidazole	6 w	*St. intermedius*	Cryptogenic	11 w	Recovery	[36]
4	57	M	Acute	C6-T1	+	ND (M + S)	Sd	N/D	Myelotomy, DR	Vancomycin	6 w	*MRSA*	Cryptogenic	N/D	Recovery	[37]
5	53	M	Acute	CM	+	ND (M + S)	5 d	Urgent	N/D	N/D	N/D	*St. milleri,* *S. intermedius.*	Cryptogenic	4 m	Recovery	[13]
6	42	M	Acute	C7	+	ND (M)	5 d	N/D	Laminectomy, DR	Linezolid	2 w	*MRSA*	Hematogenous (IE)	N/D	Recovery	[22]
7	21	M	Subacute	T12-L2	+	ND (M + S)	N/D	N/D	Myelotomy, EEC, IAC (Penicillin & Gentamicin	Penicillin IV, Flucloxacillin IV, Gentamicin IV	N/D	*S. epidermidis*	Contiguous (Epidermoid tumor)	4 m	Recovery	[38]
8	52	M	Subacute	L1	N/D	ND (M + S)	N/D	N/D	DR	Trimethoprim Sulfamethoxazole Imipenem-Cilastin (After Antibiogram: Trimethoprim-Sulfamethoxazole Minocycline	1 Y	*Nocardia sp.*	Cryptogenic	16 m	Recovery	[39]
9	25	F	Subacute	C5-C6	+	ND (M + S)	3 w	N/D	Myelotomy, DR	Isoniazid, Rifampin, Myambutol Pyrazinamide	7 w	*M. tuberculosis*	Hematogenous (Brown Sequard syndrome; Tuberculosis & SLE)	40 d (posthospital adm)	Recovery	[40]
10	56	M	Subacute	C3-C4	+	ND (M + S)	S d	N/D	N/D	Amikacin, Ceftazidime, Ciprofloxacin	3 m	*E. coli*	Hematogenous	34 m	Recovery	[41]
11	70	M	Subacute	C4-C5	+	ND (M + S)	3	N/D	Yes (N/S)	Ceftriaxone, Gentamicin, Amoxicillin PO	6 w, 2 w, 3 m	*Viridans group Streptococcus*	Hematogenous (IE, radiotherapy)	3 m	Recovery	[23]
12	59	M	Subacute	C7-T1	+	ND (M + S)	1 w	9 d	N/D	Ampicillin, Ceftriaxone, Cefpirome, Ampicillin PO	2 m	*Sterile*	Cryptogenic (Chronic sinusitis)	2 m	Recovery	[9]
13	51	M	Subacute	T2-MO	+	ND (M + S)	1 w	Urgent	N/D	Meropenem, Vancomycin, Steroid-Pulse Therapy & Immunoglobulin IV	4 w, 3 d, 3 d	*St. viridans*	Hematogenous (Dental procedure)	3 m	Recovery	[42]
14	22	M	Chronic	T12-L1	+	ND (M + S)	>2 m	Urgent	Myelotomy, DU, DR	Yes (N.S.)	N/D	*S. aureus*	Contiguous (spinal anesthesia)	40 d	Recovery	[43]
15	82	M	Chronic	T6-T7	+	ND (M + S)	4 m	N/D	N/D	Steroids, Gentamicin, Ciprofloxacin IV, Ciprofloxacin IM	10 w, 4 w, 2 m	*E. coli*	Hematogenous (UTI—diabetes)	3 m	Recovery	[33]
16	28	M	Chronic	T11	+	ND (M + S)	6 m	N/D	Y (N/S)	N/D	N/D	*N/A*	Cryptogenic (Infection)	N/D	Recovery	[34]
17	67	M	Chronic	T10-T11	+	ND (M + S)	1 Y	1 Y	SDAVF embolization	Dexamethasone Meropenem IV, Moxifloxacin PO (Alone from day 71)	4 d, 112 d	*E. faecalis*	Hematogenous (SDAVF)	N/D	Recovery	[44]
18	44	M	Chronic	T3	+	ND (M + S)	3 m	N/D	N/D	Amphotericin B Itraconazole	N/D, 3 m	*H. capsulatum*	Hematogenous (CNS histoplasmosis)	1 m	Recovery	[45]
19	52	M	N/D	C4-C5	+	ND (M + S)	N/D	N/D	DR	Oxacillin	N/D	*S. epidermiditis*	Direct inoculation (penetrating trauma) Wooden Foreign Body (WBS)	8 w	Recovery	[46]
20	55	M	N/D	Cervical	+	ND (M)	Sw	Urgent	Laminectomy	N/D—(Steroids, Amphotericin)	N/D	*C. immitis*	Hematogenous (Disseminated coccidioidomycosis)	2 m	Recovery	[47]

**Legend**: sex: F—female, M—male; location: C—cervical, T—thoracic, L—lumbar, S—sacral; infl.—inflammation; symptoms: M—motor, ND—neurological deficits, S—sensory; duration: D—day, M—months, Y—years, SW—several weeks; others: CM—conus medullaris, DR—drainage, DU—durotomy, EST—excision sinus tract, EAC—excision of abscess cavity, EEC—excision of epidermoid cyst, IAC—irrigation of abscess cavity, N/D—no data, N/S—not specified.

**Table 2:** Radiological features (myelography, CT, MRI) of intramedullary spinal cord abscess (ISCA).

Myelography (n = 8)
Spinal cord widening and swelling
CSF flow obstruction
Tethering of conus
**CT with myelography or MRI (n = 7)**
Segmental widening and swelling of cord
Complete or partial obstruction of CSF flow
**MRI (n = 50)**
Segmental widening and swelling of spinal cord
Ring-enhancing margin (abscess)
Cystic lesion with ring enhancement
CSF flow obstruction

**Table 3:** Residual group—summary of the clinical data, patient demographics, clinical manifestations, duration, microbiology, interventions and therapies, and outcomes of contemporary case reports on intramedullary spinal cord abscess (ISCA) in the current literature: systemic review of literature (1949–2022).

No.	Age	Sex	Onset	Location	Infl.	Symptoms		Symptoms to Treatment	Neurosurgical Management	Antibiotics		Pathogen	MOA	Follow-Up	Outcome	Ref.
						Name	Duration			Name	Duration					
1	72	M	Acute	C6-T2	+	ND (M + S)	5 d	Urgent	Myelotomy, EAC	Penicillin	6 w	*St. viridans*	Cryptogenic (spinal cord ependymoma)	6 w	Survived; residual ND	[25]
2	59	M	Acute	C3-C7	+	ND (M + S)	6 d	N/D	Myelotomy, DR	Cefepime IV	6 w	*St. viridans*	Cryptogenic (Plausible infection & long immobility)	N/D	Survived; residual ND	[24]
3	44	F	Acute	C2-C5	N/D	ND (M + S)	4 d	5 d	Myelotomy, DU	Meropenem, Vancomycin, Ceftriaxone	N/D	*S. milleri*	Cryptogenic	1 m	Survived; residual ND	[27]
4	35	M	Acute	C2-C5	+	ND (M + S)	2 d	2 d	Myelotomy, DR	Azithromycin, Ceftriaxone, Vancomycin,	1 m	*St. pneumoniae*	Hematogenous (Sickle cell disease)	12 d	Survived; residual ND	[61]
5	47	M	Subacute	T11	N/D	ND (M + S)	3 w	Urgent	Myelotomy, DU, DR, IAC	N/D	N/D	*St. anginosus*	Contiguous (Intrathecal morphine pump)	20 m	Survived; residual ND	[50]
6	82	F	Subacute	T12-L1	+	ND (M + S)	3 w	N/D	Laminectomy	Meropenem IV, Trimethoprim–Sulfamethoxazole IV	6 w	*N. cyriacigeorgica*	Cryptogenic	7 w	Survived; residual ND	[54]
7	77	M	Subacute	L5-S1	+	ND (M)	10 d	11 d	Laminectomy, drainage	Oxacillin, Clindamycin	N/D	*S. aureus*	Cryptogenic (Possibly arthritis)	3 m	Survived; residual ND	[56]
8	42	F	Chronic	T12	+	ND (M + S)	3.5 m	2 d	Laminectomy	Sulphonamides, N.S.	4 m	*S. aureus*	Hematogenous spread (Urinary tract infection)	9 m	Survived; residual ND	[6]
9	40	F	Chronic	T12-L2	+	ND (M + S)	6 m	N/D	Myelotomy, DU, DR	Streptomycin, Doxycycline, Rifampicin	2 m	*B. melitensis*	Hematogenous (Consumption: unpasteurized goat’s milk (Brucellosis risk factors)	2 y	Survived; residual ND	[49]
10	65	M	Chronic	T11-CA	+	ND (M + S)	2 m	N/D	N/D	Linezolid	2 w	*MRSA*	Hematogenous (IE)	1 y	Survived; residual ND	[51]
11	66	M	N/D	T5-T6	+	ND (M + S)	N/D	N/D	N/D	Metronidazole Clindamycin	6 w	*B. fragilis*	Hematogenous (?) (spondylodiscitis)	6 w	Survived; residual ND	[55]
12	45	F	Acute	C2-C6	+	ND (M + S)	2 w	N/D	N/D	Cefuroxime	6 w	*E. coli*	Hematogenous (Sepsis, UTI)	N/D	Survived; residual ND	[28]

**Legend**: sex: F—female, M—male; location: C—cervical, T—thoracic, L—lumbar, S—sacral; infl.—inflammation; symptoms: M—motor, ND—neurological deficits, S—sensory; duration: D—day, W—weeks, M—months, Y—years, SW—several weeks; others: CM—conus medullaris, DR—drainage, DU—durotomy, EAC—excision of abscess cavity, IAC—irrigation of abscess cavity, N/D—no data.

**Table 4:** Persistent group—summary of the clinical data, patient demographics, clinical manifestations, duration, microbiology, interventions and therapies, and outcomes of contemporary case reports on intramedullary spinal cord abscess (ISCA) in the current literature: systemic review of literature (1949–2022).

No.	Age	Sex	Onset	Location	Infl.	Symptoms		Symptoms to Treatment	Neurosurgical Management	Antibiotics		Pathogen	MOA	Follow-Up	Outcome	Ref.
						Name	Duration			Name	Duration					
1	71	M	Acute	C3-C6	+	ND (M + S)	5 d	N/D	IAC	Penicillin IV Chloramphenicol IV, Penicillin PO	5 w	*H. influenzae, Viridans Streptococci*	Cryptogenic	3 m	Survived; persistent ND	[14]
2	50	M	Acute	C4-C7	+	ND (M + S)	N/D	N/D	Myelotomy	Cefoperazone IV Ciprofloxacin IV	8 w	*P. cepacia*	Cryptogenic (IV drug use)	8 w	Survived; persistent ND	[58]
3	72	M	Acute	C6-T2	+	ND (M + S)	5 d	Urgent	Myelotomy, EAC	Penicillin	6 w	*St. viridans*	Cryptogenic (spinal cord ependymoma)	6 w	Survived; residual ND	[25]
4	73	M	Acute	T10-T11 & CM	+	ND (M)	N/D	3 d	N/D	Ampicillin IV	3 m	*L. monocytogenes*	Hematogenous spread (alcoholism, sepsis)	9 m	Survived; persistent ND	[32]
5	55	M	Acute	T3-T7		ND (M)	2 d	3 d	ND	Ampicillin IV	24 d	*L. monocytogenes*	Hematogenous spread (alcoholism, sepsis)	18 m	Survived; persistent ND	[32]
6	37	M	Acute	T8-T9	+	ND (M + S)	6 w	Urgent	Myelotomy	Antituberculosis Treatment PO & IV	N/D	*Sterile*	Cryptogenic	2 m	Survived; persistent ND	[29]
7	23	F	Acute	T11-T12	+	ND (M)	N/D	3 w	Laminectomy, NA	Isoniazid, Rifampicin, Pyrazinamide	N/D	*M. tuberculosis*	Cryptogenic	N/D	Survived; persistent ND	[62]
8	22	F	Acute	Holocord	+	ND (M + S)	N/D	N/D	Myelotomy, radical debulking	N/D	N/D	*S. aureus*	Cryptogenic	1 y	Survived; persistent ND	[63]
9	47	F	Acute	C7-T11	N/D	ND (M + S)	1 w	Urgent	Myelotomy	Yes (N/S)	N/D	*Oral flora*	Hematogenous (Oral infection)	N/D	Survived; Persistent, ND	[64]
10	27	M	Acute	C4-C5	+	ND (M + S)	1 w	N/D	Myelotomy, DR	Yes (N/S)	N/D	*E. coli*	Hematogenous (CKD, systemic infection)	N/D	Survived; Persistent, ND	[17]
11	61	M	Acute	T10-T11	+	ND (M + S)	1 m	N/D	Myelotomy, IE	N/D	1 m	*N/D*	Hematogenous (Systemic infection—diabetes)	35 d	Survived; Persistent, ND	[1]
12	72	M	Acute	C5, T6-T7	+	ND (M + S)	1 w	Urgent	Myelotomy, DR, DU	Ceftriaxone, Vancomycin, Ampicillin, Metronidazole, Penicillin G	6 w	*St. anginosus*	Cryptogenic	4 w	Survived, persistent ND	[12]
13	42	M	Subacute	C4-C5	+	ND (M)	15 d	2 d	Myelotomy	Penicillin IM, Chloramphenicol	4 w	*K. pneumoniae Streptococcus* sp.	Contiguous spread (Stab wound)	8 m	Survived, persistent ND	[15]
14	20	F	Subacute	L4-L5	+	ND (M + S)	N/D	N/D	EST	Gentamicin IV, Flucloxacillin IV, Metronidazole IV	N/D	*Anaerobic Streptococci*	Contiguous (dermal sinus tract, previous meningitis; prior resection of lumbar meningocele)	5 m	Survived; persistent ND	[38]
15	26	M	Subacute	L1-L4	+	ND (M + S)	N/D	N/D	Myelotomy, IAC	Penicillin, Chloramphenicol, Metronidazole	5 m	*Gram (-) bacilli*	Cryptogenic (spina bifida occulta; prior excision of intradural lipoma)	3.5 y	Survived; persistent ND	[18]
16	42	M	Subacute	C1-T3	+	ND (M)	3 w	Urgent	Myelotomy, DR	Bristopen Peflacine	N/D	*S. aureus*	Cryptogenic (IV drug use)	5 m	Survived; persistent ND	[65]
17	33	M	Subacute	T12-L3	+	ND (M + S)	3 m	N/D	Biopsy	Pipellacillin-Sodium Erythromycin	6 m	*Actinomyces*	Cryptogenic	N/D	Survived; persistent ND	[48]
18	28	M	Subacute	Cervical-MO	+	ND (M + S)	3 w	Urgent	Laminectomy, DR	Broad-Spectrum Antimicrobials	N/D	*St. viridans*	Contiguous	N/D	Survived; persistent ND	[20]
19	78	M	Subacute	T9	+	ND (M + S)	14 d	10	Myelotomy, DU, DR	Ampicillin, Cefotaxime	N/D	*L. monocytogenes*	Cryptogenic	2 m	Persistent, ND	[21]
20	65	F	Subacute	CMJ-T1	+	ND (M + S)	2 w	Urgent	Myelotomy, DR, DU	Ceftriaxone, Vancomycin, Metronidazole, Penicillin G, Meropenem, Linezolid	6 w	*St. anginosus*	Cryptogenic	16 m	Survived, persistent ND	[51]
21	69	M	Subacute	C2-C7	+	ND (M + S)	N/D	N/D	N/D	Ampicillin IV Gentamicin	12 w, 14 w	*L. monocytogenes*	Cryptogenic (Spinal stenosis with spinal cord stenosis)	2 m	Survived; persistent ND	[66]
21	61	M	Chronic	T9	N/D	ND (M + S)	4 m	1 d	Myelotomy, EAC	Yes (Not Specified)	N/D	*Sterile*	Cryptogenic	1 y	Survived; persistent ND	[67]
22	55	F	Chronic	T1-T2	+	ND (M + S)	N/D	N/D	Myelotomy, IAC	Vancomycin IV, Ceftazidime IV Clindamycin PO Ciprofloxacin PO	2/6 w	*Sterile*	Cryptogenic	N/D	Survived; persistent ND	[4]
23	68	M	Chronic	T1-T2	+	ND (M + S)	N/S	N/S	N/D	Cefotaxime IV	6 w	*Gam (-) bacilli*	Cryptogenic	3 y	Survived; persistent ND	[4]
24	19	M	Chronic	CM	N/D	ND (M + S)	3 m	N/D	Myelotomy	N/D	N/D	*Gram (+) cocci*	Cryptogenic	1 y	Persistent, ND	[17]
25	72	M	Chronic	Thoracic-Lumbar	+	ND (M + S)	5 d	N/D	Laminectomy, DR	Tazobactam/Piperacillin (Hospitalization Onset, No More Info About Drugs)	~1 Y	*K. pneumoniae*	Hematogenous (Diabetes mellitus, recurrent liver abscess)	~1 y	Persistent ND	[57]
26	32	M	N/D	C3-C6	+	ND (M + S)	N/D	14 d	Myelotomy	Ampicillin IV, Chloramphenicol IV Ampicillin PO	4 w	*L. monocytogenes*	Hematogenous spread (Alcoholism, sepsis)	18 m	Survived; persistent ND	[59]
27	50	M	N/D	C5-C7	+	ND (M)	N/D	N/D	Laminectomy, myelotomy, DR	Gentamicin, Fucidin Pefloxacin	N/D	*S. aureus*	Contagious (Epidural abscess induced septic thrombophlebitis of the veins of the spinal cord—leading to venous infarction & abscess of the spinal cord)	N/D	Survived; persistent ND	[68]
28	30	F	Subacute	T3-T7	_-	ND (M + S)	1 m	N/D	Laminectomy, myelotomy, EAC	ATT therapy	>6 m	*M. tuberculosis*	Hematogenous (Pulmonary Tuberculosis)	7 m	Survived, persistent ND	[69]

**Legend**: sex: F—female, M—male; location: C—cervical, T—thoracic, L—lumbar, S—sacral; infl.—inflammation; symptoms: M—motor, ND—neurological deficits, S—sensory; duration: D—day, M—months, Y—years, SW—several weeks; others: CM—conus medullaris, CMJ—craniocervical junction, DR—drainage, DU—durotomy, EST—excision sinus tract, EAC—excision of abscess cavity, IAC—irrigation of abscess cavity, N/D—no data, N/S—not specified.

**Table 5:** Death group—summary of the clinical data, patient demographics, clinical manifestations, duration, microbiology, interventions and therapies, and outcomes of contemporary case reports on intramedullary spinal cord abscess (ISCA) in the current literature: systemic review of literature (1949–2022).

No.	Age	Sex	Onset	Location	Infl.	Symptoms		Symptoms to Treatment	Neurosurgical Management	Antibiotics		Pathogen	MOA	Follow-Up	Outcome	Ref.
						Name	Duration			Name	Duration					
1	76	M	Acute	T3-T12	+	ND (M + S)	N/D	N/D	N/D	Yes (Not Specified)	3 w	*Gram (-) bacilli*	Hematogenous spread (Septic embolus, pyelonephritis)	3 w	Died	[19]
2	51	M	Acute	Cervical	+	ND (M + S)	N/D	N/D	N/D	N/D	N/D	*Staphylococcus*	Hematogenous spread (Alcoholism, bronchopneumonia)	N/D	Died	[52]
3	59	M	Acute	C4-C6	+	ND (M + S)	N/D	N/D	Myelotomy	Chloramphenicol IV Ceftazidime IV Metronidazole	31 d	*B. disiens*	Hematogenous spread (bronchiectasis)	31 d	Died	[53]
4	59	M	Subacute	C3-T1	+	ND (M + S)	2 w	Urgent	Laminectomy, DR	Amikacin, Ceftriaxone Trimethoprim Sulfamethoxazole	12 d	*N. asteroides*	Hematogenous spread (Cerebral abscess, diabetes)	N/D	Died	[70]
5	79	M	Acute	C3-C4	+	ND (M + S)	S d	N/D	N/D	IV Trimethoprim-Sulfamethoxazole, Dexamethasone	10	*N. farcinica*	Hematogenous spread	N/D	Died	[30]
6	45	F	Acute	C2-C6	+	ND (M + S)	2 w	N/D	N/D	Cefuroxime	6 w	*E. coli*	Hematogenous spread (Sepsis, UTI)	N/D	Died	[28]
7	19	M	N/D	T12-L1	N/D	ND (M + S)	Sw	N/D	Laminectomy, USG—guided aspiration	Voriconazole	N/D	*Aspergillus*	Contagious (Vertebral discitis, osteomyelitis)	N/D	Died	[26]
8	69	M	Subacute	T7-CM	+	ND (M + S)	17 d	N/D	N/D	Ceftriaxone, Metronidazole—On the 3rd day of admission. Meropenem—On the 5th day.	1 w	*B. pseudomallei*	Cryptogenic (suspicion of hematogenous, (Diabetes mellitus)	N/D	Died	[71]
9	50	M		C2-C6		ND (S)			myelotomy	N/D	N/D	*L. monocytogenes*	Hematogenous spread (sepsis)	N/D	Died	[72]
10	44	F	Chronic	T10-T11	+	ND (M + S)	>1 Y	N/D	Laminectomy, DR	N/D	N/D	*C. albicans*	Hematogenous spread (History of CNS fungal infection and neurotuberculosis)	N/D	N/D	[60]

**Legend**: sex: F—female, M—male; location: C—cervical, T—thoracic, L—lumbar, S—sacral; infl.—inflammation; Symptoms: M—motor, ND—neurological deficits, S—sensory; duration: D—day, M—months, Y—years, SW—several weeks; others: CM—conus medullaris, DR—drainage, EEC—excision of epidermoid cyst, IAC—irrigation of abscess cavity, N/D—no data, N/S—not specified.

## Data Availability

Not applicable.

## Supplementary Materials

The following supporting information can be downloaded at: https://www.mdpi.com/article/10.3390/jcm11175148/s1, Table S1: The mechanism behind the development of intramedullary spinal cord abscess in adults; Table S2: The predisposing factors of intramedullary spinal cord abscess in adults (see Table 1, Table 2 and Table 3 for additional information); Table S3: Concomitant diseases.
