# Supplementary material for: Intramedullary Spinal Cord Abscess with Concomitant Spinal Degenerative Diseases: A Case Report and Systematic Literature Review
Source: J Clin Med. 2022 Aug 31;11(17):5148. doi: 10.3390/jcm11175148 (PMC9457049; doi:10.3390/jcm11175148)
Supplement: Supplementary file 1 [file jcm-11-05148-s001.zip › jcm-1818119-supplementary.pdf]

Journal of
Clinical Medicine

MDPI

Supplementary materials

Supplementary Table S1. The mechanism behind the development of intramedullary spinal cord abscess in adults.

Mechanism of infection	N (%)
Cryptogenic spread	32 (45.7%)
Contagious spread	7 (10%)
Hematogenous spread	22 (31.4%)
Penetrating trauma	4 (5.7%)

Supplementary Table S2. The predisposing factors of intramedullary spinal cord abscess in adults (see Tables 1–3 for additional information).

Predisposing factors	N (%)
Spondylodiscitis and vertebral osteomyelitis/discitis	4 (5.7%)
Anatomical abnormalities of the spinal cord and/or vertebral column	4 (5.7%)
Diabetes mellitus with systemic effects	4 (5.7%)
Spinal dysraphism	3 (4.2%)
Urinary tract infection	3 (4.2%)
Infective endocarditis	3 (4.2%)
Sepsis originating from either E. coli, S. aureus, or Gram (-) bacilli	3 (4.2%)
Spinal anaesthesia	2 (2.8%)
Postoperative complications (Spinal cord dermoid cyst & ependymoma)	2 (2.8%)
Tuberculosis and SLE	2 (2.8%)
Oral infections and dental procedures	2 (2.8%)
Dermal sinus tract	1 (1.4%)
Epidural abscesses	1 (1.4%)
Intrathecal morphine pump	1 (1.4%)
Cervical spinal stenosis	1 (1.4%)
Pyelonephritis	1 (1.4%)
Bronchopneumonia	1 (1.4%)
bronchiectasis	1 (1.4%)
Brucellosis	1 (1.4%)

Chronic kidney diseases	1 (1.4%)
Disseminated coccidioidomycosis	1 (1.4%)
Histoplasmosis	1 (1.4%)
Neurotuberculosis	1 (1.4%)

Supplementary Table S3. Concomitant diseases.

Concomitant diseases	N (%)
Diabetes and concomitant systemic infection	5 (7.1%)
Heroin/Alcohol addiction	5 (7.1%)
Urinary tract infection	4 (5.7%)
Vertebral infection (osteomyelitis, discitis and spondylodiscitis, arthritis)	4 (5.7%)
Sepsis and septic emboli	3 (4.2%)
Spinal anatomical abnormalities	3 (4.2%)
Pulmonary diseases	2 (2.8%)
Tuberculosis	2 (2.8%)
Oral infection	2 (2.8%)
Infective endocarditis	2 (2.8%)
Disseminated coccidioidomycosis	2 (2.8%)
Chronic kidney diseases	1 (1.4%)
Chronic sinusitis	1 (1.4%)
Systemic lupus erythematosus	1 (1.4%)
Sickle cell disease	1 (1.4%)
Pulmonary arteriovenous fistula	1 (1.4%)
CNS histoplasmosis	1 (1.4%)
Ulcerative colitis	1 (1.4%)
